# Supplementary material for: Semi-Site-Specific Primer PCR: A Simple but Reliable Genome-Walking Tool
Source: Curr Issues Mol Biol. 2023 Jan 5;45(1):512–23. doi: 10.3390/cimb45010034 (PMC9857434; doi:10.3390/cimb45010034)
Supplement: Supplementary file 1 [file cimb-45-00034-s001.zip › cimb-2134562-supplementary.pdf]

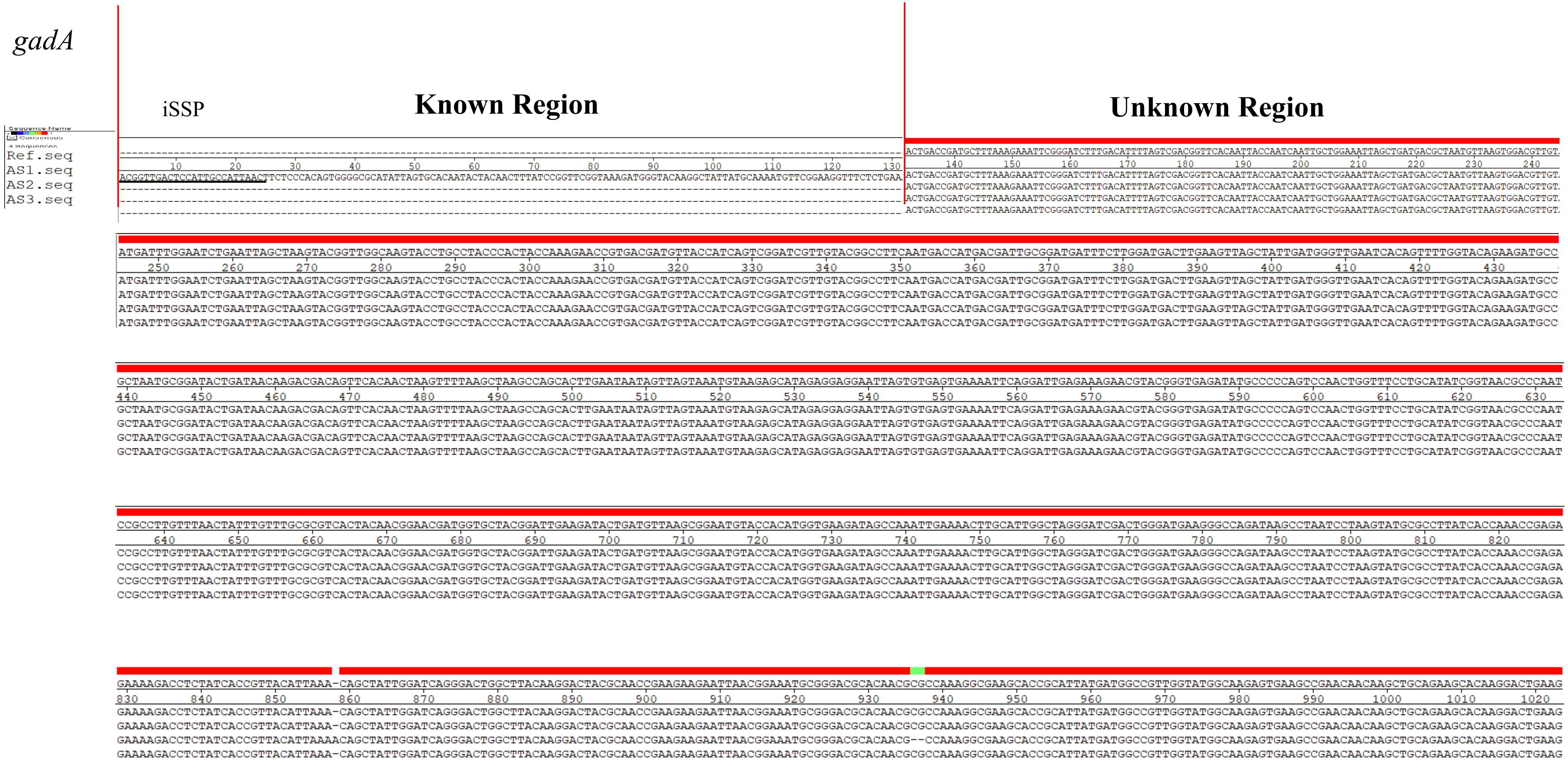
gadA
iSSP
Known Region
Unknown Region
Ref.seq
AS1.seq
AS2.seq
AS3.seq

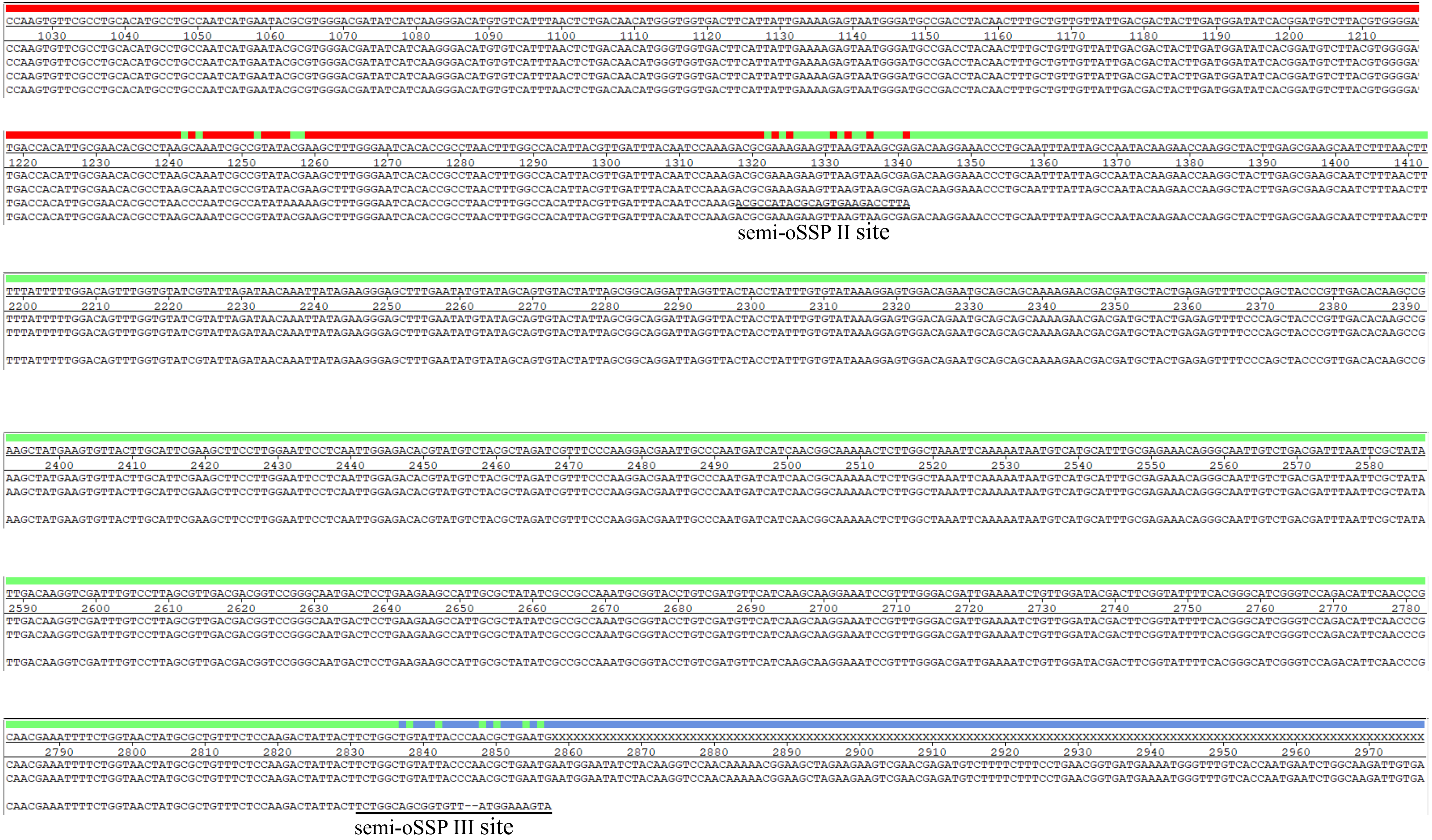
semi-oSSP II site
semi-oSSP III site

```
XXXXXXXXXXXXXXXXXXXXXXXXXXXXXXXXXXXXXXXXXXXXXXXXXXXXXXXXXXXXXXXXXXXXXXXXXXXXXXXXXXXXXXXXXXXXXXXXXXXXXXXXXXXXXXXXXXXXXXXXXXXXXXXXXXXXXXXXXXXXXXXXXXXXXXXXXXXXXXXXXXXXXXXXXXXXXXXXXXXXXXXXXXXXXXXXX
  6780      6790      6800      6810      6820      6830      6840      6850      6860      6870      6880      6890      6900      6910      6920      6930      6940      6950      6960      6970
CTTGGCTTGGACCGATGACAAGAACGATCCACAATACGAAATTTCAAAAGAAAACTTGGATATTTTGGAAAACGCAACGGATGCTAAGGGCCGTAAGATCAAGGTCGTTAAGCTCTACTTGCCAAAGCCAATCACCATTACCAAGGAAGAAAGCGAAGGCGTTGACGCTGTTGATGGGACCTTACCACGGACCGA
CTTGGCTTGGACCGATGACAAGAACGATCCACAATACGAAATTTCAAAAGAAAACTTGGATATTTTGGAAAACGCAACGGATGCTAAGGGCCGTAAGATCAAGGTCGTTAAGCTCTACTTGCCAAAGCCAATCACCATTACCAAGGAAGAAAGCGAAGGCGTTGACGCTGTTGATGGGACCTTACCACGGACCGA
```

```
XXXXXXXXXXXXXXXXXXXXXXXXXXXXXXXXXXXXXXXXXXXXXXXXXXXXXXXXXXXXXXXXXXXXXXXXXXXXXXXXXXXXXXXXXXXXXXXXXXXXXXXXXXXXXXXXXXXXXXXXXXXXXXXXXXXXXXXXXXXXXXXXXXXXXXXXXXXXXXXXXXXXXXXXXXXXXXXXXXXXXXXXXXXXXXXXX
       6980      6990      7000      7010      7020      7030      7040      7050      7060      7070      7080      7090      7100      7110      7120      7130      7140      7150      7160
AGGCGAACGCTTAGCTGCTAGCTACGTGAACTACTACACGGCTAATGGCGGCATTGTCTTCCCACTGTTCGGCGACCCAGCCGATGAGAAGGCCAAAGCAACACTGGAAGAACTTTACCCAGACCGCAAAGTTGTGGGCGTTCCCGCTCGTGAAGTCTTACTCGGTGGCGGTAATATCCACTGCATTACGCAACA
AGGCGAACGCTTAGCTGCTAGCTACGTGAACTACTACACGGCTAATGGCGGCATTGTCTTCCCACTGTTCGGCGACCCAGCCGATGAGAAGGCCAAAGCAACACTGGAAGAACTTTACCCAGACCGCAAAGTTGTGGGCGTTCCCGCTCGTGAAGTCTTACTCGGTGGCGGTAATATCCACTGCATTACGCAACA
```

```
XXXXXXXXXXXXXXXXXXXXXXXXXXXXXXXXXXXXXXXXXXXXXXXXXXXXXXXXXXXXXXXXXXXXXXXXXXXXXXXXXXXXXXXXXXXXXXXXXXXXXXXXXXXXXXXXXXXXXXXXXXXXXXXXXXXXXXXXXXXXXXXXXXXXXXXXXXXXXXXXXXXXXXXXXXXXXXXXXXXXXXXXXXXXXXXX
  7170      7180      7190      7200      7210      7220      7230      7240      7250      7260      7270      7280      7290      7300      7310      7320      7330      7340      7350      7360
AGTTCCTGAAGCTTAATCGGGAAATGTGAAAGGTCAGGTGAAGAGCCTGGCCTTTTCTGTTAAAAATAAGGAGTGAATCCATCATGACAAAACGTAAAATTGTAGTTGCATTAGGCGGAAACGCCATTTTAGCTGACGATGCTTCGGCCGCTGCCCAACAGGCCGCTCTGGCTGACACAGCGAAGTCACTGGTCC
AGTTCCTGAAGCTTAATCGGGAAATGTGAAAGGTCAGGTGAAGAGCCTGGCCTTTTCTGTTAAAAATAAGGAGTGAATCCATCATGACAAAACGTAAAATTGTAGTTGCATTAGGCGGAAACGCCATTTTAGCTGACGATGCTTCGGCCGCTGCCCAACAGGCCGCTCTGGCTGACACAGCGAAGTCACTGGTCC
```

```
XXXXXXXXXXXXXXXXXXXXXXXXXXXXXXXXXXXXXXXXXXXXXXXXXXXXXXXXXXXXXXXXXXXXXXXXXXXXXXXXXXXXXXXXXXXXXXXXXXXXXXXXXXXXXXXXXXXXXXXXXXXXXXXXXXXXXXXXXXXXXXXXXXXXXXXXXXXXXXXXXXXXXXXXXXXXXXXXXXXXXXXXXXXXXXXX
       7370      7380      7390      7400      7410      7420      7430      7440      7450      7460      7470      7480      7490      7500      7510      7520      7530      7540      7550
AGTTCGTCAAGCAAGGCGACCAGCTGATCATTTCTCACGGGAACGGGCCGCAAGTCGGCAACCTGCTGTTACAACAGGCCGCTGGCAGTACGGCTAAGAACCCGGCCATGCCATTGGATACGGCTGTGGCCATGACGCAAGGGAGCATTGGCTACTGGTTGGAAAACGCCATGGATAAAGCCTTGGCTGATGAAG
AGTTCGTCAAGCAAGGCGACCAGCTGATCATTTCTCACGGGAACGGGCCGCAAGTCGGCAACCTGCTGTTACAACAGGCCGCTGGCAGTACGGCTAAGAACCCGGCCATGCCATTGGATACGGCTGTGGCCATGACGCAAGGGAGCATTGGCTACTGGTTGGAAAACGCCATGGATAAAGCCTTGGCTGATGAAG
```

```
XXXXXXXXXXXXXXXXXXXXXXXXXXXXXXXXXXXXXXXXXXXXXXXXXXXXXXXXXXXXXXXXXXXXXXXXXXXXXXXXXXXXXXXXXXXXXXXXXXXXXXXXXXXXXXXXXXXXXXXXXXXXXXXXXXXXXXXXXXXXXXXXXXXXXXXXXXXXXXXXXXXXXXXXXXXXXXXXXXXXXXXXXXXXXXXX
  7560      7570      7580      7590      7600      7610      7620      7630      7640      7650      7660      7670      7680      7690      7700      7710      7720      7730      7740      7750
GCCTAACGAAGGATGTTGCCACAGTAGTCACCCAAGTTGAAGTGGCACCCGATGACCCAGCCTTTACGGATCCTAGCAAGCCGATCGGGCCATTCTATACAGAAGCCGAAGTCGCTACGGAAAAGCAAGCTCATCCAGACAATGTCTACGTGGAAGATGCGGGTCGGGGATACCGGCGTGTGGTAGCTTCCCCCA
GCCTAACGAAGGATGTTGCCACAGTAGTCACCCAAGTTGAAGTGGCACCCGATGACCCAGCCTTTACGGATCCTAACAAGCCGATCGGGCCATTCTATACAGAAGCCGAAGTCGCTACGGAAAAGCAAGCTCATCCAGACAATGTCTACGTGGAAGATGCGGGTCGGGGATACCGGCGTGTGGTAGCTTCCCCCA
```

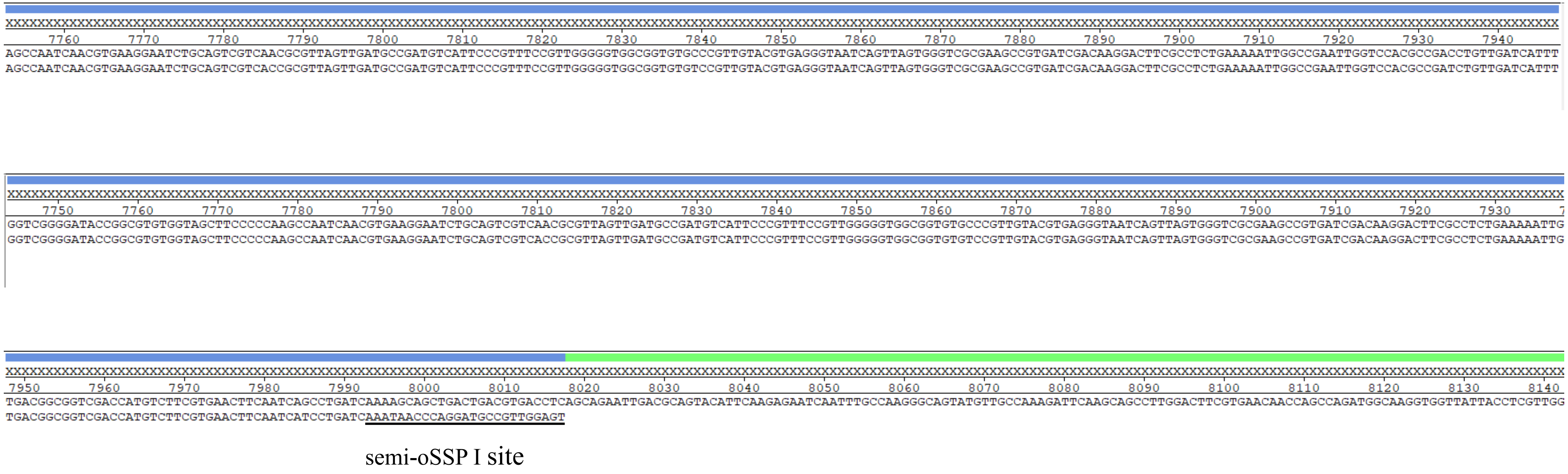
semi-oSSP I site

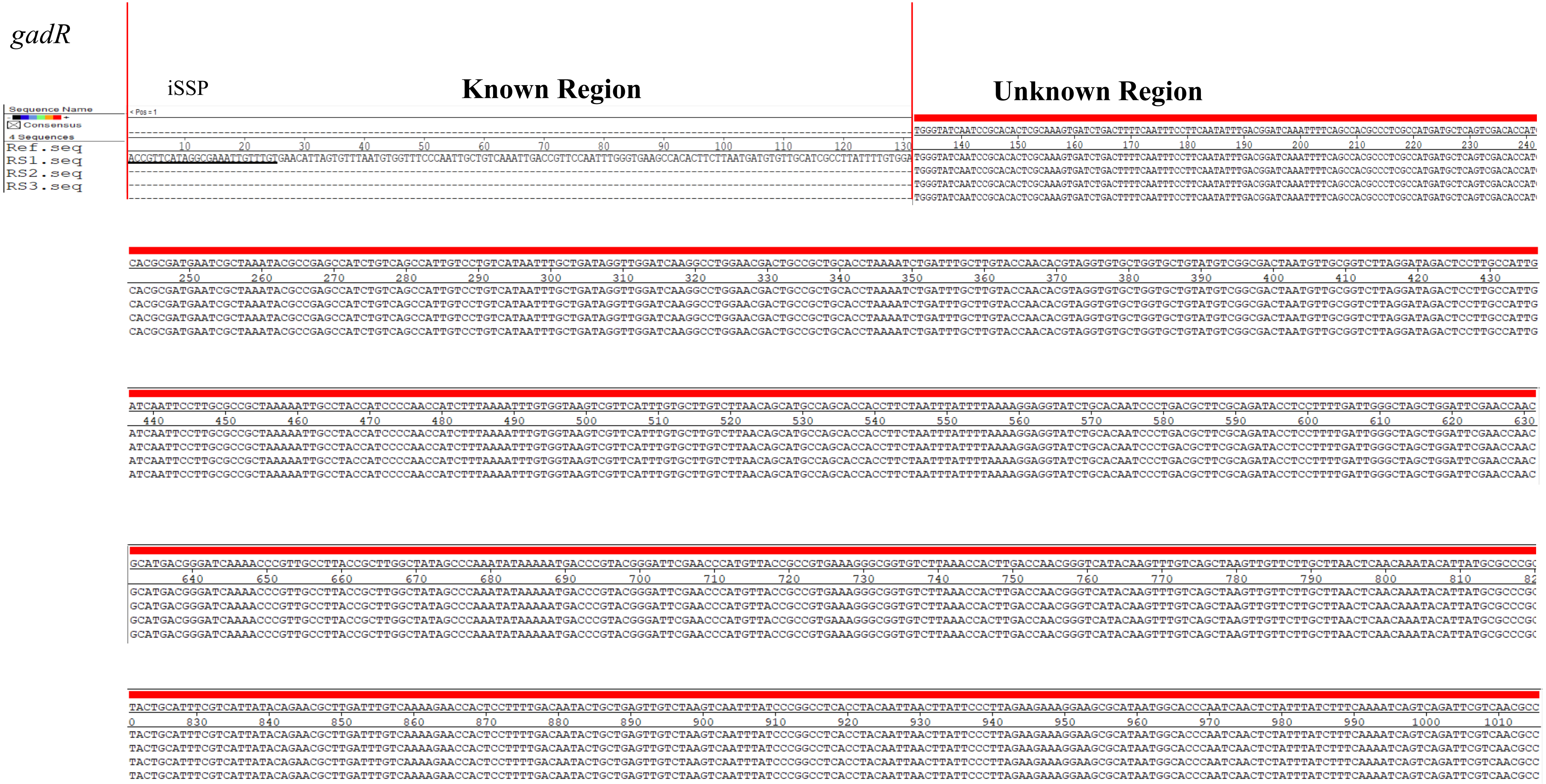
gadR
iSSP
Known Region
Unknown Region
Sequence Name
Consensus
4 Sequences
Ref.seq
RS1.seq
RS2.seq
RS3.seq

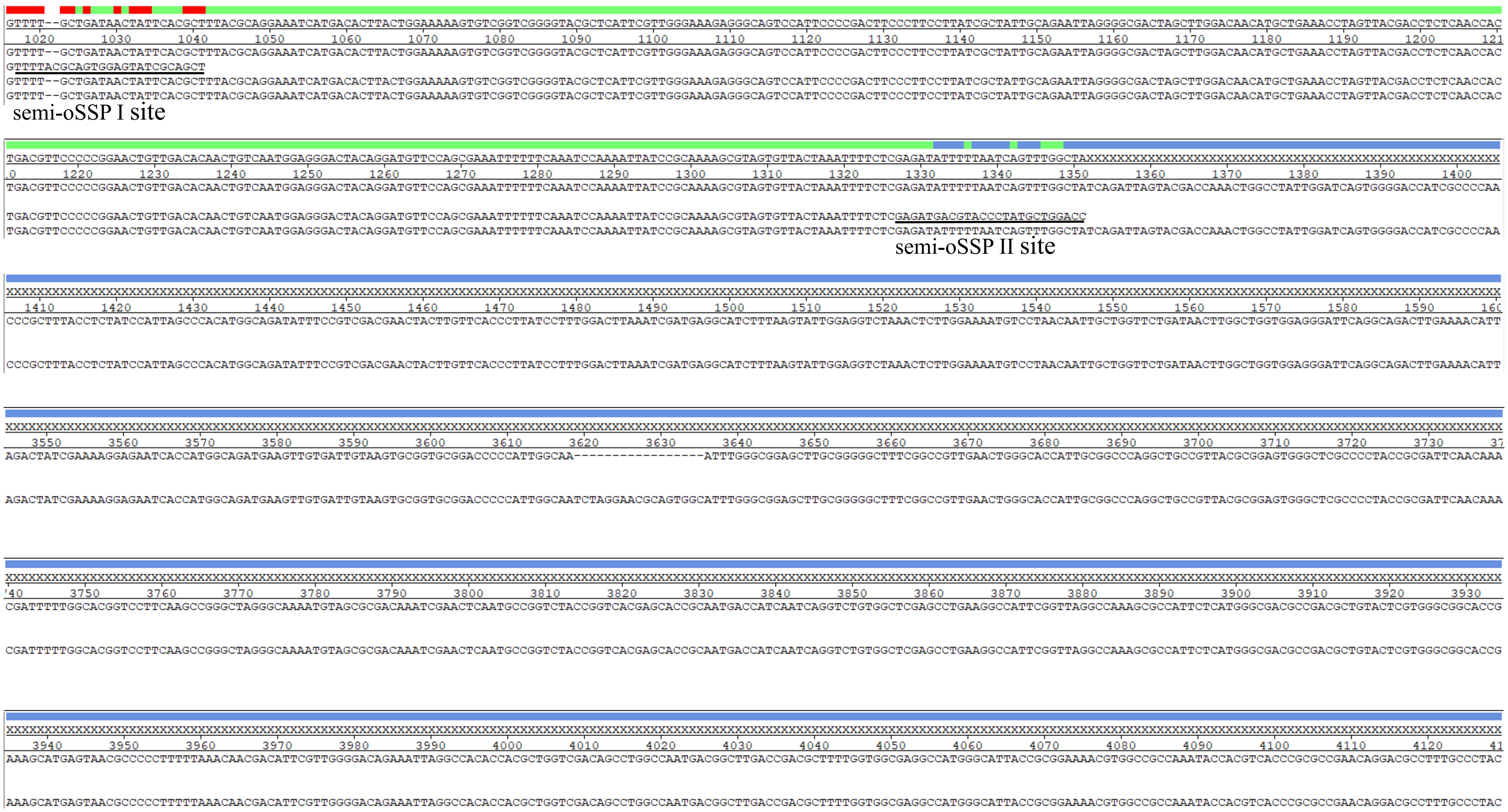
semi-oSSP I site
semi-oSSP II site

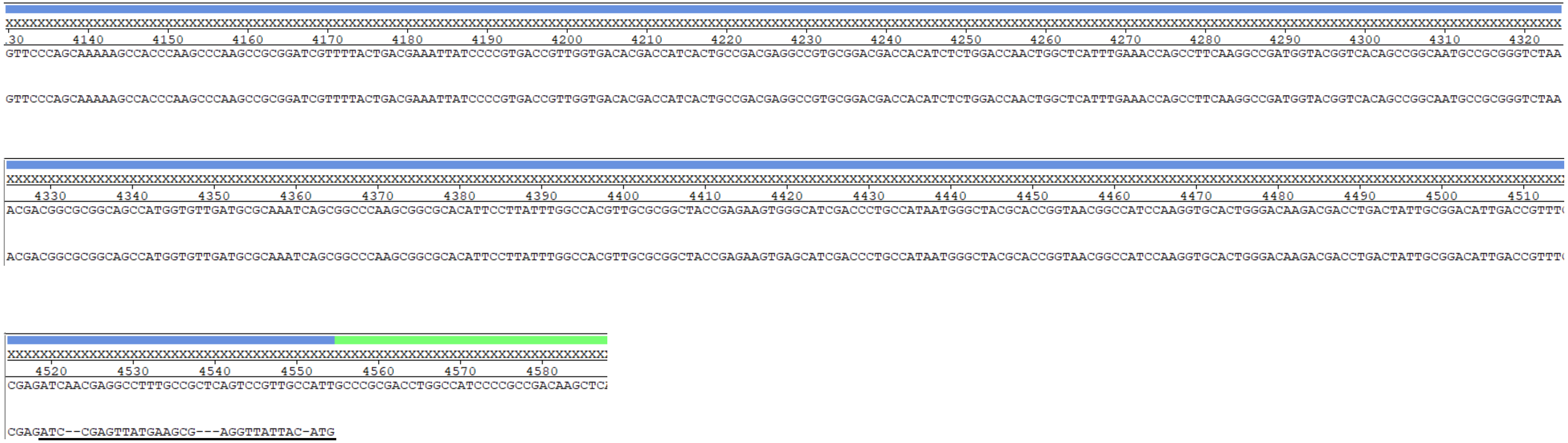

semi-oSSP III site

hyg

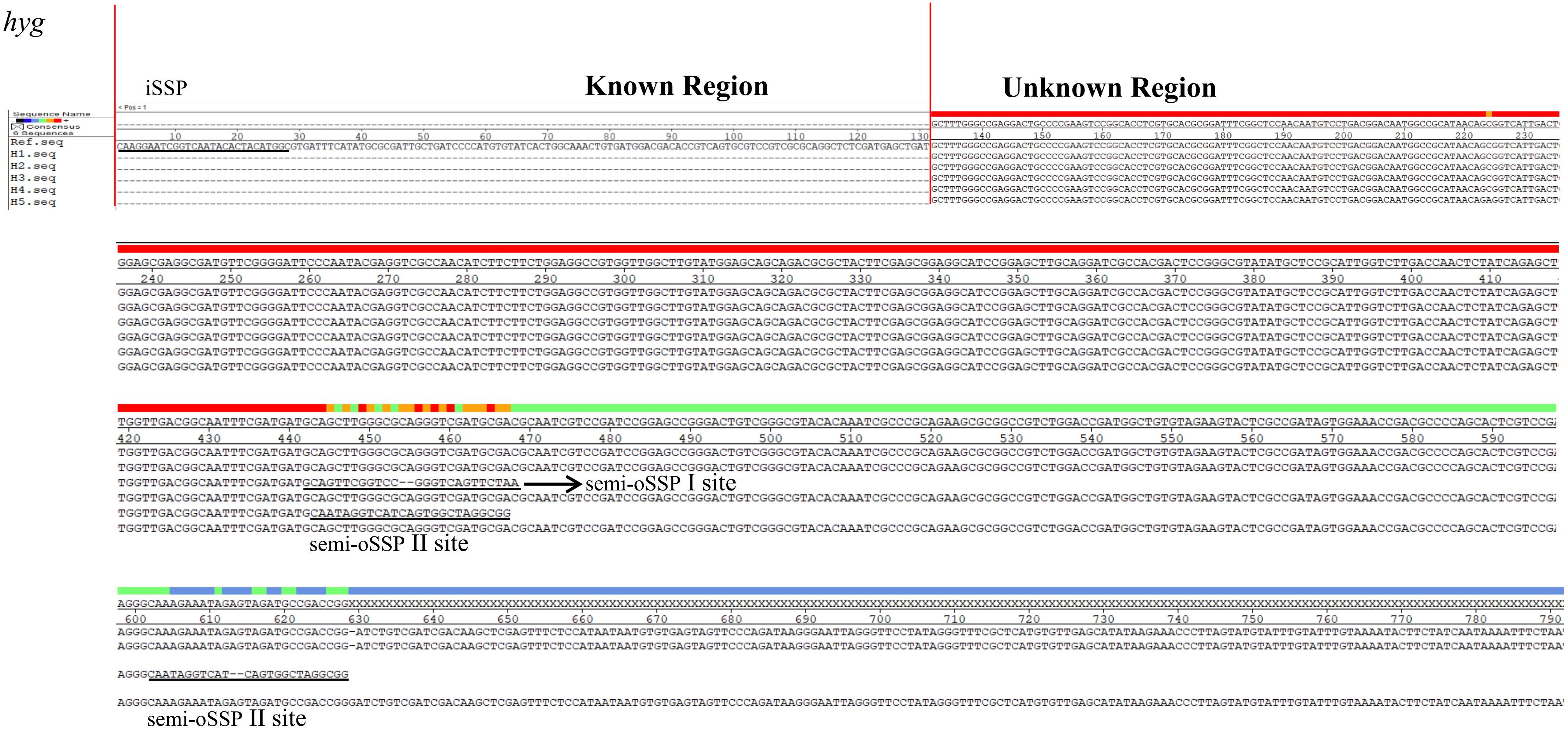
iSSP
Known Region
Unknown Region
Ref.seq
H1.seq
H2.seq
H3.seq
H4.seq
H5.seq
semi-oSSP I site
semi-oSSP II site
semi-oSSP II site

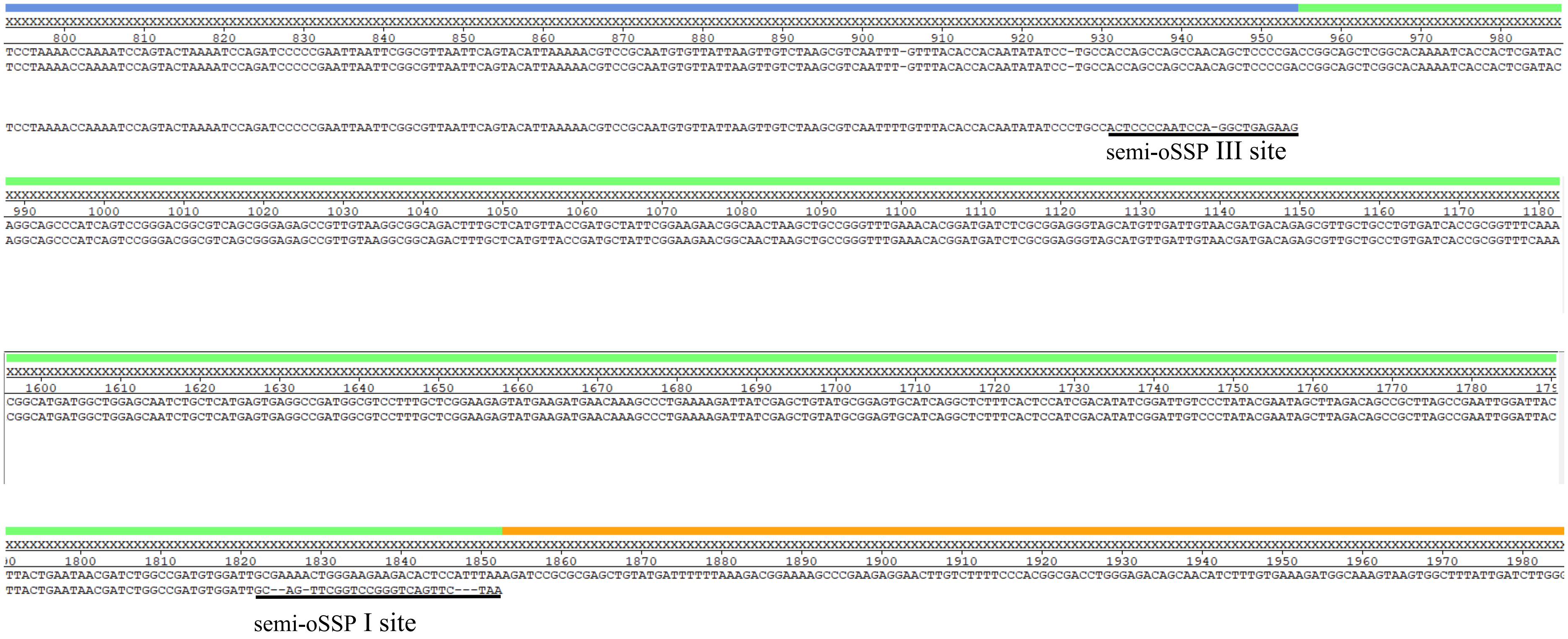
semi-oSSP III site
semi-oSSP I site
